# Supplementary material for: Post-Viral Fatigue Following SARS-CoV-2 Infection during Pregnancy: A Longitudinal Comparative Study
Source: Int J Environ Res Public Health. 2022 Nov 26;19(23):15735. doi: 10.3390/ijerph192315735 (PMC9737157; doi:10.3390/ijerph192315735)
Supplement: Supplementary file 1 [file ijerph-19-15735-s001.zip › File S1. 05_06_FADIGA_1_1_V_27_05.pdf]

ZIKAlliance Estudo post-COVID-19 Formulário de Registro de Caso (FRC) **Fad-FRC 1.1 SEGUIMENTO**

**AVALIAÇÃO PARA SINTOMAS DE FADIGA – VISITA SUBSEQUENTE**

**DATA:______________IG___________________**

**IDENTIFICAÇÃO:___________________________________________________________**

**Questionário - Cansaço/Fadiga**

**1. Você tem problemas com cansaço intenso, fadiga ou exaustão persistente ou recorrente (que não vai embora ou sempre volta)?**

☐ Sim

☐ Não (se não, reponda Questão 1ª e termine o questionario)

☐ Persistência de outros sintomas de COVID? Quais_____________________________________

**1a. Você teve fadiga após o coronavirus?**

☐ Não

☐ Sim, mas não sinto mais. Senti por mais ou menos ___ dia(s) ___ mes(es)

**2. Quando você começou a sentir esse cansaço ou fadiga?**

☐ Há menos de um mês atrás

☐ De 1 a menos de 2 meses atrás

☐ De 2 a menos de 3 meses atrás

☐ Já sentia antes da última visita

**3. Sintomas iniciaram aproximadamente** __ __ (dia)__ __(mês) __ __ (ano)

**4. Este cansaço profundo ou fadiga acontece somente porque você tem feito muita atividade e melhora quando você descansa?**

☐ Sim

☐ Não

**5. Este cansaço intenso, exaustão, ocorre por causa de uma doença de longa duração que você tem?**

☐ Sim

☐ Não

☐ Não sei

**5a. Escreva no espaço abaixo doença(s) que causa(m) seus sintomas de cansaço intenso:**

____________________________________________________

**6. Esse cansaço ou fadiga aparece com que frequência?**

a.☐ Ocasionalmente, de vez em quando.

b.☐ Frequentemente, menos de 50% do tempo.

**c.☐ Mais do que 50% do tempo.**

**7. Esse cansaço/fadiga afeta (dificulta) suas atividades pessoais, sociais, de trabalho, ou em família?**

a.☐ Não, de jeito nenhum.

b.☐ Um pouco, mas eu ainda posso fazer a maioria das coisas que
eu costumava fazer normalmente.

**c.☐ Eu precisei parar pelo menos algumas das minhas atividades.**

**d.☐ Eu não posso mais fazer as atividades que eu costumava fazer antes.**

**8. Em relação às suas atividades, que você é capaz de fazer agora comparado com o que você costumava fazer antes de adoecer?**

**a.☐ Consigo fazer 50% ou menos do que eu fazia antes.**

b.☐ Consigo fazer mais de 50% ou as mesmas coisas que eu fazia antes.

As perguntas seguintes são relacionadas a como você vem se sentindo nos 7 últimos dias, incluindo hoje

**9. Que nota você daria para a fadiga ou cansaço que você está sentindo? Dê nota zero para nenhuma fadiga, e dez para a pior fadiga que você pode imaginar. ___ ___**

**10. Que nota você daria para a fadiga ou cansaço físico (do corpo) que você está sentindo? Dê nota zero para nenhuma fadiga física, e dez para a pior fadiga física que você pode imaginar. ___ ___**

**11. Que nota você daria para a fadiga ou cansaço mental que você está sentindo? Dê nota zero para nenhuma fadiga mental, e dez para a pior fadiga mental que você pode imaginar. ___ ___**

**12. Que nota você daria para a dor que você está sentindo? Dê nota zero para nenhuma dor, e dez para a pior dor que você pode imaginar. ___ ___**

**13. Comparado com a última avaliação, e em relação aos seus sintomas de cansaço, voce considera que está?**

☐ Não tinha o sintoma na última visita

☐ Totalmente recuperado

☐ Muito melhor

☐ Um pouco melhor

☐ O mesmo

☐ Um pouco pior

☐ Muito pior

**14- Parte B: Informação sobre resultado do COVID**

a. PCR - SARS-CoV-2: ☐ Positivo ☐ Negativo ☐ Não Realizado ☐ Não disponível

b. Sorologia – SARS-CoV-2: ☐ Positivo ☐ Negativo ☐ Não Realizado ☐ IgG ☐ IgM ☐ IgA

**Resultado FADIGA**

**-** Se a triagem for positiva para **Fadiga Significativa**, pela **primeira vez**, completar **Fad-FRC2** (questionário de fadiga adicional)

**-** Se o participante já foi triado para **Fadiga Significativa** em **consulta anterior**, aplicar direto o questionário **Fad-FRC2.1** (questionário de fadiga adicional seguimento)

***Fadiga significativa requer seguintes respostas para TODAS as questões acima:***

1= **Sim** 4= **Não** 5= **Não** 6= **c**; 7= **c** ou **d**; 8= **a**

☐ caso **positivo** para fadiga significante – aplicar o questionário ☐ Fat-CRF 2 ou ☐ CRF 2.1

☐ caso **negativo** para fadiga significante

**Questionário preenchido por:**

**Nome:_______________________________________________________**

**Função:_______________________________Data___________________**
