# Supplementary material for: Post-Viral Fatigue Following SARS-CoV-2 Infection during Pregnancy: A Longitudinal Comparative Study
Source: Int J Environ Res Public Health. 2022 Nov 26;19(23):15735. doi: 10.3390/ijerph192315735 (PMC9737157; doi:10.3390/ijerph192315735)
Supplement: Supplementary file 1 [file ijerph-19-15735-s001.zip › File S2. 05_06_FADIGA_1_V_27_05.pdf]

ZIKAlliance Estudo post-COVID-19 Formulário de Registro de Caso (FRC) **Fad-FRC 1**

AVALIAÇÃO INICIAL PARA SINTOMAS DE FADIGA

DATA:_______________IG____________________

IDENTIFICAÇÃO:____

Questionário - Cansaço/Fadiga

1. Você tem problemas com cansaço intenso, fadiga ou exaustão persistente ou recorrente (que não vai embora ou sempre volta)?

☐ Sim

☐ Não (se não, questionário termina aqui)

☐ Persistência de outros sintomas de COVID? Quais_____________________________________

2. Este problema iniciou (ou se tornou muito pior) após a suspeita de coronavírus (cansaço intenso, fadiga ou exaustão)?

☐ Sim

☐ Não, o problema já existia antes do coronavírus

☐ Não, o problema iniciou depois de eu me sentir completamente bem depois do coronavírus

3. Quando você começou a sentir esse cansaço ou fadiga?

☐ Há menos de um mês atrás

☐ De 1 a menos de 3 meses atrás

☐ De 3 a menos de meses atrás

☐ Há mais de 6 meses

☐ Tenho este problema por toda minha vida, não me lembro sentir-me bem

3a. Sintomas iniciaram aproximadamente

__ __ (dia) __ __ (mês) __ __ (ano)

4. Este cansaço ou fadiga acontece somente porque você tem feito muita atividade e melhora quando você descansa?

☐ Sim

☐ Não

5. Este cansaço intenso, exaustão, ocorre por causa de uma doença de longa duração que você tem?

☐ Sim

☐ Não

☐ Não sei

5a. Escreva no espaço abaixo doença que causa seus sintomas de cansaço intenso:

____

6. Esse cansaço ou fadiga aparece com que frequência?

a.☐ Ocasionalmente, de vez em quando.

b.☐ Frequentemente, menos de 50% do tempo.

c.☐ Mais do que 50% do tempo.

7. Esse cansaço/fadiga afeta (dificulta) suas atividades pessoais, sociais, de trabalho, ou em família?

a.☐ Não, de jeito nenhum.

b.☐ Um pouco, mas eu ainda posso fazer a maioria das coisas que
eu costumava fazer normalmente.

c.☐ Eu precisei parar pelo menos algumas das minhas atividades.

d.☐ Eu não posso mais fazer as atividades que eu costumava fazer antes.

8. Em relação às suas atividades, que você é capaz de fazer agora comparado com o que você costumava fazer antes de adoecer?

a.☐ Consigo fazer 50% ou menos do que eu fazia antes.

b.☐ Consigo fazer mais de 50%, ou as mesmas coisas que eu fazia antes.

9. Você tem alguma doença séria (física ou mental) que necessita tratamento médico? a. ☐ Sim

b. ☐ Não

c. Qual(is)? ____

As perguntas seguintes são relacionadas a como você vem se sentindo nos 7 últimos dias, incluindo hoje

10. Que nota você daria para a fadiga ou cansaço que você está sentindo? Dê nota zero para nenhuma fadiga, e dez para a pior fadiga que você pode imaginar. ___ ___

11. Que nota você daria para a fadiga ou cansaço físico (do corpo) que você está sentindo? Dê nota zero para nenhuma fadiga física, e dez para a pior fadiga física que você pode imaginar. ___ ___

12. Que nota você daria para a fadiga ou cansaço mental que você está sentindo? Dê nota zero para nenhuma fadiga mental, e dez para a pior fadiga mental que você pode imaginar. ___ ___

13. Que nota você daria para a dor que você está sentindo? Dê nota zero para nenhuma dor, e dez para a pior dor que você pode imaginar. __ __

14- Parte B: Informação sobre resultado do COVID

a. PCR - SARS-CoV-2: ☐ Positivo ☐ Negativo ☐ Não Realizado ☐ Não disponível

b. Sorologia – SARS-CoV-2: ☐ Positivo ☐ Negativo ☐ Não Realizado ☐ IgG ☐ IgM ☐ IgA

Se a triagem for positiva para Fadiga Significativa, completar Fad-FRC2

Fadiga significativa requer seguintes respostas para **TODAS** as questões acima:

1= **Sim** 4= **Não** 5= **Não** 6= **c**; 7= **c** ou **d**; 8= **a**

☐ caso **positivo** para fadiga significante – aplicar o questionário Fat-CRF 2

☐ caso **negativo** para fadiga significante

Questionário preenchido por:

Nome:____

Função:________________________________**Data**____________________
