# Supplementary material for: Post-Viral Fatigue Following SARS-CoV-2 Infection during Pregnancy: A Longitudinal Comparative Study
Source: Int J Environ Res Public Health. 2022 Nov 26;19(23):15735. doi: 10.3390/ijerph192315735 (PMC9737157; doi:10.3390/ijerph192315735)
Supplement: Supplementary file 1 [file ijerph-19-15735-s001.zip › File S3. 05_06_FADIGA_2_1_V_27_05.pdf]

ZIKAlliance Estudo post-COVID-19 Formulário de Registro de Caso (FRC) **Fad-FRC 2.1**

# QUESTIONÁRIO ADICIONAL DE FADIGA SEGUIMENTO/ACOMPANHAMENTO
# VISITA SUBSEQUENTE

**DATA:______________IG___________________**

**IDENTIFICAÇÃO:___________________________________________________________**

**1. Você teve alguns dos sintomas, descritos a seguir, com muita frequência, ou de forma persistente ou recorrente, desde que ficou doente? Se você tem qualquer um destes sintomas, responda como eles estão de acordo com o seguinte esquema:**

1- Sintomas leves ou só de vez em quando
2- Sintomas moderados e frequentes – incomodam
3- sintomas intensos ou graves – incomodam muito

| **Você tem esses sintomas?** | **NÃO** | **SIM - Intensidade** | | |
|---|---|---|---|---|
| | | **leve** | **Moderado** | **Intenso/grave** |
| **1**-dor de garganta | | | | |
| **2**-sintomas de gripe | | | | |
| **3**-febre ou calafrios | | | | |
| **4**-gânglios inflamados no pescoço ou axilas | | | | |
| **5**- intolerância nova alimentos, remédios, produtos químicos, cheiros, ou outros produtos | | | | |
| **6**-mal estar após atividades ou esforço, que demora mais para melhorar (mais de 24h) | | | | |
| **7**-dor após atividade ou esforço, que demora para melhorar (mais de 24h) | | | | |
| **8**-dor nos músculos | | | | |
| **9**-espasmos ou câimbras nos músculos | | | | |
| **10**-dor em duas ou mais articulações, sem vermelhidão ou calor | | | | |
| **11**-dor nas articulações que migra para outras articulações, sem vermelhidão ou calor | | | | |
| **12**- falta de ar, dificuldade para respirar ou fôlego curto para fazer atividades ou ao esforço | | | | |
| **13**-fraqueza nos músculos | | | | |
| **14**-sensibilidade anormal à luz ou barulho | | | | |
| **15**-desequilíbrio ou instabilidade quando fica em pé ou incapacidade de focar a visão | | | | |
| **16**-falta de coordenação, ou falta de firmeza nos movimentos (falta de firmeza para caminhar) | | | | |
| **17**-problemas de memória recente | | | | |
| **18**- problemas de concentração | | | | |
| **19**-confusão | | | | |
| **20**-desorientação | | | | |
| **21**-dificuldade para compreender as coisas ou pensar com clareza | | | | |
| **22**-dificuldade para encontrar as palavras ao falar | | | | |
| **23**-dificuldade para guardar ou lembrar uma informação | | | | |
| **24**-pensamento ou raciocínio lentificado | | | | |
| **25**-sono que não é repousante e revigorante | | | | |
| **26**-problemas na qualidade ou duração do sono, como insônia, trocar o dia pela noite, acordar no meio da noite | | | | |
| **27**-fadiga ou exaustão mental ou física intensos após mínimo esforço, que persistem por muito tempo (mais que 24h) | | | | |
| **28**-piora dos sintomas após esforço ou atividades, que persiste por muito tempo (mais que 24h) | | | | |
| **29**-piora dos sintomas com stress | | | | |

| Você tem esses sintomas? | NÃO | SIM - Intensidade | | |
|---|---|---|---|---|
| | | leve | Moderado | Intenso/grave |
| **30**- intolerância (ou dificuldade) para ficar em pé | | | | |
| **31**-tontura ou sensação de desmaio quando fica em pé | | | | |
| **32**-palpitações (sensação de que seu coração está acelerado) quando fica em pé | | | | |
| **33**-palpitações em outros momentos | | | | |
| **34**-sensação de tontura | | | | |
| **35**-palidez extrema | | | | |
| **36**-sudorese extrema | | | | |
| **37**-intolerância a extremos de calor ou frio | | | | |
| **38**-náusea, enjôo | | | | |
| **39**-sensação de intestino irritado (diarréia, constipação, dor abdominal, gazes) | | | | |
| **40**-problemas na bexiga, como urgência para urinar mais frequente que o normal, ou acordar à noite para urinar | | | | |
| **41**-apetite anormal ou mudança de peso não intencional | | | | |
| **42**-não sente cheiros | | | | |
| **43**-não sente gosto | | | | |
| **44**-tosse | | | | |

**2-Desde sua última avaliação, você foi diagnosticado com alguma doença adicional que requer tratamento médico?**

☐ Sim, Quai(s)?_________________________________________________________________

☐ Não

**3-Qual é o seu peso atual em Kg?____________**

**4-Quais medicações você tomou nos últimos 3 meses?**

___________________________________________________________________________________________
___________________________________________________________________________________________
___________________________________________________________________________________________

**5- Quais medicações você está tomando atualmente?**

___________________________________________________________________________________________
___________________________________________________________________________________________
___________________________________________________________________________________________

**Questionário preenchido por:**

**Nome:_______________________________________________________**

**Função:_______________________________Data___________________**
