# Supplementary material for: Post-Viral Fatigue Following SARS-CoV-2 Infection during Pregnancy: A Longitudinal Comparative Study
Source: Int J Environ Res Public Health. 2022 Nov 26;19(23):15735. doi: 10.3390/ijerph192315735 (PMC9737157; doi:10.3390/ijerph192315735)
Supplement: Supplementary file 1 [file ijerph-19-15735-s001.zip › File S4. 05_06_FADIGA_2_V_27_05.pdf]

ZIKAlliance Estudo post-COVID-19 Formulário de Registro de Caso (FRC) **Fad-FRC 2**

QUESTIONÁRIO ADICIONAL DE FADIGA

DATA:_______________IG____________________

IDENTIFICAÇÃO:____

Responda às questões 1 a 5 em relação ao período desde que você desenvolveu os sintomas de fadiga

1.O seu cansaço piora com esforço ou atividade mínima, e esta piora dura por bastante tempo (geralmente por mais de 24 horas; continua no dia seguinte e pode durar vários dias)?

☐ Não, ou de vez em quando

☐ Frequentemente, mas menos de metade (50%) do tempo

☐ **Mais que 50% do tempo (maior parte do tempo)**

2. Você sente dores que não tinha antes de adoecer, como dor muscular, nas articulações (juntas) ou dor de cabeça?

☐ Não, ou de vez em quando

☐ Frequentemente, mas menos de metade (50%) do tempo

☐ **Mais que 50% do tempo (maior parte do tempo)**

3. Você tem problemas de sono que não tinha antes de adoecer, como acordar se sentindo como se não tivesse dormido , dificuldade em pegar no sono, ou ficar acordado durante a noite

☐ Não, ou de vez em quando

☐ Frequentemente, mas menos de metade (50%) do tempo

☐ **Mais que 50% do tempo (maior parte do tempo)**

4. Você acha que está com raciocínio lento ou difícil, tem dificuldade de prestar atenção ou de se concentrar, ou tem confusão mental, que você não tinha antes de adoecer?

☐ Não, ou de vez em quando

☐ Frequentemente, mas menos de metade (50%) do tempo

☐ **Mais que 50% do tempo (maior parte do tempo)**

5. Você tem dificuldade de ficar em pé parado por mais tempo que alguns segundos ou minutos, porque se sente mal, por exemplo com tonturas, batimento rápido do coração, enjoo ou fraqueza, que você não tinha antes de adoecer?

☐ Não, ou de vez em quando

☐ Frequentemente, mas menos de metade (50%) do tempo

☐ Mais que 50% do tempo (maior parte do tempo)

6. Você teve alguns dos sintomas descritos a seguir com muita frequência, ou de forma persistente ou recorrente, desde que ficou doente? Se você tem qualquer um destes sintomas, responda como eles estão de acordo com o seguinte esquema:

1- Sintomas leves ou só de vez em quando

2- Sintomas moderados e frequentes – incomodam

3- sintomas intensos ou graves – incomodam muito

Você tem esses sintomas?	NÃO	SIM - Intensidade		
		leve	Moderado	Intenso/grave
**1**-dor de garganta				
**2**-sintomas de gripe				
**3**-febre ou calafrios				
**4**-gânglios inflamados no pescoço ou axilas				
**5**- intolerância nova alimentos, remédios, produtos químicos, cheiros, ou outros produtos				
**6**-mal estar após atividades ou esforço, que demora mais para melhorar (mais de 24h)				
**7**-dor após atividade ou esforço, que demora para melhorar (mais de 24h)				
**8**-dor nos músculos				
**9**-espasmos ou câimbras nos músculos				
**10**-dor em duas ou mais articulações, sem vermelhidão ou calor				
**11**-dor nas articulações que migra para outras articulações, sem vermelhidão ou calor				
**12**- falta de ar, dificuldade para respirar ou fôlego curto para fazer atividades ou ao esforço				
**13**-fraqueza nos músculos				
**14**-sensibilidade anormal à luz ou barulho				
**15**-desequilíbrio ou instabilidade quando fica em pé ou incapacidade de focar a visão				
**16**-falta de coordenação, ou falta de firmeza nos movimentos (falta de firmeza para caminhar)				
**17**-problemas de memória recente				
**18**- problemas de concentração				
**19**-confusão				
**20**-desorientação				
**21**-dificuldade para compreender as coisas ou pensar com clareza				
**22**-dificuldade para encontrar as palavras ao falar				
**23**-dificuldade para guardar ou lembrar uma informação				
**24**-pensamento ou raciocínio lentificado				
**25**-sono que não é repousante e revigorante				
**26**-problemas na qualidade ou duração do sono, como insônia, trocar o dia pela noite, acordar no meio da noite				
**27**-fadiga ou exaustão mental ou física intensos após mínimo esforço, que persistem por muito tempo (mais que 24h)				
**28**-piora dos sintomas após esforço ou atividades, que persiste por muito tempo (mais que 24h)				
**29**-piora dos sintomas com stress				
**30**- intolerância (ou dificuldade) para ficar em pé				
**31**-tontura ou sensação de desmaio quando fica em pé				
**32**-palpitações (sensação de que seu coração está acelerado) quando fica em pé				
**33**-palpitações em outros momentos				
**34**-sensação de tontura				
**35**-palidez extrema				
**36**-sudorese extrema				
**37**-intolerância a extremos de calor ou frio				
**38**-náusea, enjôo				
**39**-sensação de intestino irritado (diarréia, constipação, dor abdominal, gazes)				
**40**-problemas na bexiga, como urgência para urinar mais frequente que o normal, ou acordar à noite para urinar				
**41**-apetite anormal ou mudança de peso não intencional				
**42**-não sente cheiros				
**43**-não sente gosto				
**44**-tosse				

7-Você tem alguma doença séria (física ou mental) que necessita tratamento médico?

☐ Não

☐ Sim , Qual(is)?____

8-Você tem algum dos seguintes problemas? Marque todos que se aplicam

	Hipotireoidismo (glândula tireóide funciona pouco)
	Hipertireoidismo (glâncula tireóide funciona muito)
	Câncer. Qual?_________________________
	Artrite reumatóide
	Fibromialgia
	Outra doença reumática grave como lúpus ou polimiosite
	Doença do coração, como insuficiência cardíaca
	DPOC (bronquite crônica ou enfisema) grave ou outra
	Doença respiratória grave
	Anemia grave
	Insuficiência dos rins (rins não funcionam bem)
	Diabetes
	Doença de Cushing ou Addison (glândulas adrenais não funcionam bem)
	Transtorno bipolar, esquizofrenia, depressão, anorexia ou bulimia
	Esclerose múltipla
	Doença de Parkinson, miastenia gravis, ou outra doença neurológica exceto síndrome da fadiga crônica
	Apnéia do sono/narcolepsia
	Hepatite, tuberculose ou outra doença infecciosa crônica. Qual?________________________________
	Consumo excessivo de álcool ou drogas recreativas
	Outra doença grave. Qual?____

9-Se você tem qualquer dos problemas acima, algum deles está fora de controle?

	Sim, pelo menos um deles está fora de controle
	Não, todos estão estáveis e bem controlados
	Não sei
	Não se aplica, não tenho nenhum problema

10-Qual é o seu peso atual em Kg?____________ e sua altura, em cm?________________

11-Quais medicações você tomou nos últimos 3 meses?

____

____

____

12- Quais medicações você está tomando atualmente?

____

____

____

Questionário preenchido por:

Nome:____

Função:_______________________________Data___________________
