# Supplementary material for: Post-Viral Fatigue Following SARS-CoV-2 Infection during Pregnancy: A Longitudinal Comparative Study
Source: Int J Environ Res Public Health. 2022 Nov 26;19(23):15735. doi: 10.3390/ijerph192315735 (PMC9737157; doi:10.3390/ijerph192315735)
Supplement: Supplementary file 1 [file ijerph-19-15735-s001.zip › Table S1-S2.pdf]

**Supplementary Table S1.** Frequencies of visits by study group.

| Number of visits | Group 1 (N = 259) n (%) | Group 2 (N = 131) n (%) | Group 3 (N = 198) n (%) |
|---|---|---|---|
| 1 visit only | 116 (44.8) | 73 (55.7) | 122 (61.6) |
| > 1 visit | 147 (56.8) | 58 (44.3) | 76 (38.4) |
| ≥ 3 visits | 98 (37.8) | 34 (26.0) | 45 (22.7) |

Group 1=pregnant women with COVID-19 diagnosed during antenatal care; Group 2=pregnant women with positive SARS-CoV-2 serology at delivery; Group 3=pregnant women with negative SARS-CoV-2 serology at delivery.

**Supplementary Table S2.** Comparison of fatigue and pain scores in symptomatic cases according to Visit and timing after SARS-CoV-2 infection.

| Outcome at each time point | | Group 1 N* | Group 1 Score Median (range) | Group 2 N* | Group 2 Score Median (range) | Group 3 N* | Group 3 Score Median (range) | P-value |
|---|---|---|---|---|---|---|---|---|
| **Overall fatigue score** | Delivery | 28 | 6 (2-10) | 1 | 7 | 4 | 6 (5-7) | 0.62# |
| | 6 weeks | 34 | 5 (2-8) | 2 | 8 (5-8) | 3 | 6 (5-7) | 0.24# |
| | 3 months | 18 | 7 (4-8) | 3 | 7.5 (7-8) | 2 | 6.5 (6-7) | 0.5# |
| | 6 months | 9 | 7 (2-10) | 0 | NA | 1 | 6 | NA |
| **Physical fatigue score** | Delivery | 28 | 5 (0-10) | 1 | 7 | 4 | 7 (6-7) | 0.80# |
| | 6 weeks | 34 | 6 (0-10) | 2 | 8 (8-8) | 3 | 4 (3-8) | 0.06# |
| | 3 months | 18 | 7 (0-10) | 3 | 10 | 2 | 7.5 (7-8) | 0.17# |
| | 6 months | 9 | 7 (0-10) | 0 | NA | 1 | 8 | NA |
| **Mental fatigue score** | Delivery | 28 | 5.5 (0-10) | 1 | 8 | 4 | 5 (0-7) | 0.40# |
| | 6 weeks | 34 | 7 (0-10) | 2 | 9 (9-10) | 3 | 7 (6-10) | 0.08# |
| | 3 months | 18 | 7.5 (0-10) | 3 | 8.5 (7-10) | 2 | 7.5 (6-9) | 0.73# |
| | 6 months | 9 | 8 (0-10) | 0 | NA | 1 | 6 | NA |
| **Pain score** | Delivery | 28 | 4 (0-8) | 1 | 8 | 4 | 0 (0-4) | 0.07# |
| | 6 weeks | 34 | 5 (0-10) | 2 | 10 (8-10) | 3 | 0 (0-10) | 0.06# |
| | 3 months | 18 | 5 (0-10) | 3 | 3.5 (0-7) | 2 | 0 (0-0) | 0.32# |
| | 6 months | 9 | 6 (0-10) | 0 | NA | 1 | 8 | NA |

# Kruskal-Wallis test; N*= number of cases with positive fatigue; NA= not applicable.

Group 1= pregnant women with COVID-19 diagnosed during antenatal care; Group 2=pregnant women with positive SARS-CoV-2 serology at delivery; Group 3=pregnant

women with negative SARS-CoV-2 serology at delivery. Score: from 0 as absence to 10 for the worst relating to how participant has felt over the past 7 days.
